# Assessment of Graphene Oxide and Nanoclay Based Hybrid Filler in Chlorobutyl-Natural Rubber Blend for Advanced Gas Barrier Applications

**DOI:** 10.3390/nano11051098

**Published:** 2021-04-23

**Authors:** Jibin Keloth Paduvilan, Prajitha Velayudhan, Ashin Amanulla, Hanna Joseph Maria, Allisson Saiter-Fourcin, Sabu Thomas

**Affiliations:** 1 International and Interuniversity Centre for Nanoscience and Nanotechnology, Mahatma Gandhi University, Kottayam 686560, Kerala, India; jibin@mgu.ac.in (J.K.P.); prajitha@mgu.ac.in (P.V.); 2 School of Chemical Sciences, Mahatma Gandhi University, Kottayam 686560, Kerala, India; 3 Amity Institute of Nanotechnology, Amity University, Noida 201313, Uttar Pradesh, India; ashin.jnv@gmail.com; 4 School of Energy Materials, Mahatma Gandhi University, Kottayam 686560, Kerala, India; hanna@mgu.ac.in; 5 Normandie Université, UNIROUEN, INSA Rouen, CNRS, GPM, 76000 Rouen, France

**Keywords:** rubber, nanocomposite, graphene oxide, nanoclay, carbon black, permeation, mechanical properties

## Abstract

Nanomaterials have engaged response from the scientific world in recent decades due to their exceptional physical and chemical properties counter to their bulk. They have been widely used in a polymer matrix to improve mechanical, thermal, barrier, electronic and chemical properties. In rubber nanocomposites, nanofillers dispersion and the interfacial adhesion between polymer and fillers influences the composites factual properties. In the present work, a comparison of the hybrid effects of carbon black with two different nanofillers (graphene oxide and nanoclay) was studied. The 70/30 composition of chlorobutyl rubber/natural rubber elastomer blend was taken as per the blend composition optimized from our previous studies. The hybrid effects of graphene oxide and nanoclay in dispersing the nanofillers were studied mainly by analyzing nanocomposite barrier properties. The results confirm that the combined effect of carbon black with graphene oxide and nanoclay could create hybrid effects in decreasing the gas permeability. The prepared nanocomposites which partially replace the expensive chlorobutyl rubber can be used for tyre inner liner application. Additionally, the reduction in the amount of carbon black in the nanocomposite can be an added advantage of considering the environmental and economic factors.

## 1. Introduction

Ever since the popularity of nanotechnology has grown in recent decades, nanomaterials have been widely used as a reinforcing agent in a polymer matrix to improve the overall properties of polymer composites [1]. Nanomaterials, which essentially have at least one dimension less than 100 nm and a high surface to volume ratio is a prominent substitute to conventional fillers due to significant output with lower filler loading [2,3,4].

Natural rubber (caoutchouc) is organic latex which is extensively used in many industries due to its unique chemical and mechanical properties. Natural rubber is widely exploited by tyre industries because it improves their excellent mechanical properties upon vulcanizing. Chlorobutyl rubber (CIIR) is an isobutylene-isoprene copolymer which is used to make tyre inner liners and gas masks due to its low gas permeability [5,6]. It has been reported that a blend of chlorobutyl rubber and natural rubber (NR) can significantly reduce the cost of inner liners in a tubeless tyre. Many industries use nanomaterials as fillers in rubber to improve the mechanical, barrier and thermal properties of materials [7,8].

In tyre technology, carbon black with particle size in the 15–200 nm range is widely used as a reinforcing agent to improve mechanical properties. Although there are reports on nano-silica as an alternative, the overall filler interaction with rubber matrix and the economy makes carbon black (CB) a favorite in the tyre industry [9]. Nanoclays (NC) are yet other nanomaterials that are widely used in polymer technology due to the 2D structure [10]. Many researchers have already reported that nanoclays act as gas barrier agents in polymer nanocomposites [11,12,13,14,15,16,17]. Evenly nanoclay dispersion creates a tortuous path in the polymer matrix so that it hinders the movement of gas molecules through the material [18]. Recent research shows graphene oxide (GO) can also be used in a polymer matrix to improve the mechanical and barrier properties due to its high aspect ratio and high Young’s modulus in the range of 200 GPa [19,20,21,22]. The high aspect ratio of GO can create very good interfacial interactions between GO and polymer matrix even in lower filler loading thus reducing overall polymer composites’ weight to a greater extent. The 2D structure of GO can also create a tortuous path to the gas molecule to pass through the polymer composite [16,23,24,25]. Ratnayake et al. successfully dispersed the montmorillonite into the natural rubber latex matrix and got a tremendous increase in the mechanical properties with high interfacial compatibility [26]. Yang et al. modified the graphene oxide with octadecylamine (ODA) and reinforced into brominated butyl rubber (BIIR) This modification helps to improve the dispersion of the filler in rubber matrix and results excellent tortuous diffusion pathway for enhanced barrier properties [27]. Zhong et al. improved the gas barrier properties by introducing sulfur–graphene oxide hybrids to the styrene butadiene rubber matrix. They have managed to obtain excellent interfacial interactions between the hybrid filler and the rubber matrix. This strong interface restricts the chain mobility and thereby reduces the free volume which inhibits the gas molecules to penetrate through rubber matrix [28]. Alipour et al. discussed the oxygen diffusion rate and penetrability of the polyethylene and EVOH-based multilayered films having 5- and 19-layer C-films of thicknesses 150 nm or even bigger. They have studied the crystallization behavior of the polymer layers using a synchrotron wide-angle/small-angle X-ray scattering technique and correlated these with the permeability and mechanical strength. It was evident from the work that the various multilayer films having the same oxygen diffusion ability [29]. Numerous studies were reported on hybrid filler technology in rubber, but a little has been based on GO and nanoclay hybrid rubber [26,30,31].

In the present work, we have studied the effect of nanofiller (NF) dispersion in rubber matrix with various amounts of GO and nanoclay. Additionally, we studied how the synergistic hybrid effects of CB with GO and nanoclay work in the 70/30 composition of CIIR/NR blend, which has a variety of industrial applications, including the tyre industry. This work’s prime motive is to analyze the influence of hybrid fillers in the air permeability characteristics.

## 2. Materials and Methods

### 2.1. Materials

NR was provided by the Rubber Research Institute, Kottayam, and Kerala, India. CIIR from ExxonMobil was provided kindly by Apollo Tyres Pvt Ltd., Kalamassery, Kerala, India. GO was prepared by the oxidation of 99% unadulterated graphite flakes purchased from Sigma Aldrich, Ernakulam, Kerala, India. Cloisite 10A with an average size of 1.93 nm was obtained from Southern Clay Products, Mumbai, India. Cloisite 10A is an organically modified montmorillonite with dimethyl, benzyl, and hydrogenated tallow (HT), quaternary ammonium salt (125 meq/100 g of clay, moisture <2%). Carbon black (HAF N330) was supplied by AB Brothers, Mumbai, India. All other vulcanizing agents were obtained from Apollo tyres (Kalamassery, Kerala, India).

### 2.2. Methods 

#### 2.2.1. Synthesis of Graphene Oxide

**Figure 1:**
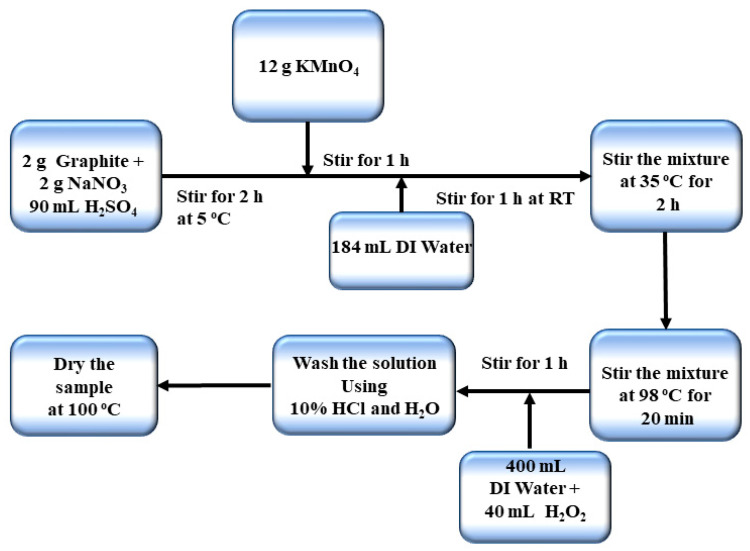
Schematic representation of graphene oxide synthesis.

Graphene oxide was synthesized by using an improved Hummers method [32].The detailed schematic of the synthesis route is explained in Figure 1. A total of 2 g of graphite, 2 g of sodium nitrate and 90 mL of sulfuric acid (98% H_2_SO_4_) were added together in a 1000 mL Florence flask and stirred for 2 h. The temperature was kept less than 20 °C. Then, 12 g of potassium permanganate was added to the solution slowly. After one hour, 184 mL of deionized water (DI) was added slowly and stirred for 1 h at room temperature. The temperature of the mixture was maintained at 35 °C and was stirred for 2 h. The temperature was increased up to 98 °C for 20 min. The heater was turned off and 40 mL of H_2_O_2_ was added to the solution by which the color changes to yellow. After adding 400 mL of DI water the solution was further stirred for 1 h. The product mixture was washed with 10% HCl and then with DI water for many times until the pH value between 6 and 7. Then it was subsequently placed in a vacuum oven and dried at 100 °C. The schematic representation of synthesis is given below.

#### 2.2.2. Preparation of CIIR/NR Blend Nanocomposites

The polymer nanocomposites were prepared through a melt mixing method on a laboratory two-roll mill [28]. NR was masticated for about two to three minutes and the masticated NR was then mixed with CIIR trailed by addition of the curing agents as per the protocol given in Table 1. Cure characteristics were deliberated by employing a Prescott’s Rheoline moving Die Rheometer (ASTMD 5289), at a temperature of 160 °C, frequency of 1.67 Hz and strain rate of 0.5° arc. The mixes were cured at their particular cure times by means of a hydraulic press under a pressure of about 120 bar at 160 °C. The formulation used for the preparation in phr is given in Table 2.

### 2.3. Characterizations

Transmission electron microscopy (TEM) is deliberated to be the utmost widespread modus operandi in characterizing nanomaterials in electron microscopy. The information about chemical constituents and imaginings of nanomaterials at a specific resolution equivalent to atomic magnitudes are provided using TEM JEM-2100 HRTEM (Musashino, Akishima, Tokyo, Japan) by means of a point to point steadfastness of 0.194 nm and a speed up voltage of 200 kV was used to evaluate the graphene oxide sheet structure. From the TEM images we can confirm the morphology of GO, how it exfoliated and the variation from graphitic structure. 

The Fourier Transform Infra Red spectrum is an active tool to elucidate both covalent and non-covalent interactions of GO. The fundamental details of graphene oxide together with polymer nanocomposites were dogged by Fourier Transform Infra Red spectroscopy (Shimadzu IR Tracer-100, Nishinokyo Kuwabara-cho, Kyoto, Japan). From the FTIR we can analyze the efficient groups responsible for the copious properties which are present in the sample and we can ascertain the establishment of hydrogen bonding. In polymer analysis, the exploration of structures, details about composition, density features, crystallinity data, magnitude of oxidative degradation, extent of functionalization and blend compatibility are common deliverables of IR spectroscopy. The Fourier transform infrared spectroscopy peaks were obtained by Shimadzu IR Tracer-100 by collecting 32 shots extending from 500 to 4000 cm^−1^, with a resolution of 0.4 cm^−1^. 

The mechanical properties of the nanocomposite were quantified by with a Tinius Olsen H 25 K-L Universal Testing machine (UTM), Surrey RH1 5DZ, UK (ASTM D412). Sample was prepared as per ASTM die C, the dumbbell shaped samples with a gauge length of 30 mm and the thickness of 2 mm were used at a testing crosshead speed of 500 mm/minute with the cell load of 1KN. We have taken the average of five readings not varying within broad limits and the results on these average values, with corresponding values of standard deviations mentioned in the graph.

Prescott’s Rheoline Moving Die Rheometer, Gloucestershire, UK was utilized to accomplish the rheological study as per the ASTM D 5283 standard at a frequency of 1.67 Hz, and 0.5 arc strain rate. Gas permeability studies were done by using Gas Permeability Tester from ATS FAAR S.p.A, Segrate, Italy, using oxygen as per the ASTM D1434-82 standard.

Prescott’s Rheoline Moving Die Rheometer, Gloucestershire, UK was utilized to accomplish the rheological study as per the ASTM D 5283 standard at a frequency of 1.67 Hz, and 0.5 arc strain rate. Gas permeability studies were done by using Gas Permeability Tester from ATS FAAR S.p.A, Segrate, Italy, using oxygen as per the ASTM D1434-82 standard.

## 3. Results and Discussions

### 3.1. Characterizations of Synthesized Graphene Oxide

#### 3.1.1. Morphological Analysis

**Figure 2:**
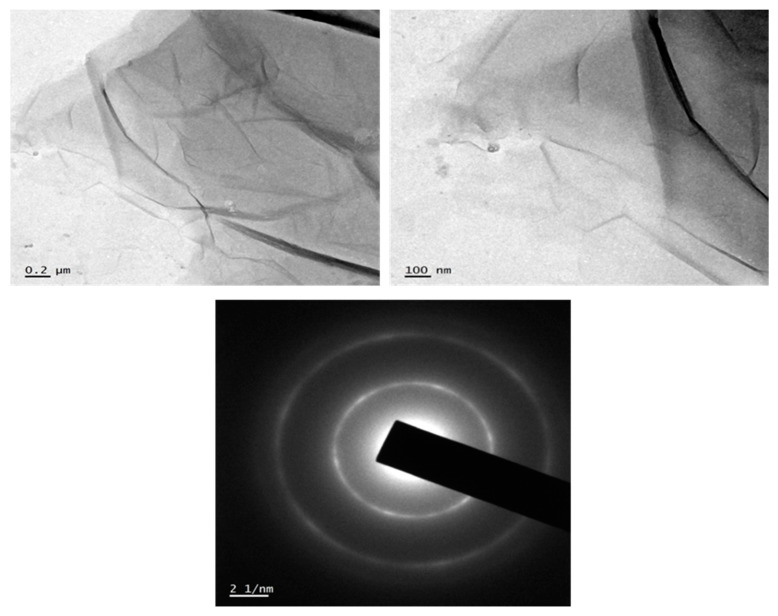
Transmission electron microscopy (TEM) images of synthesized graphene oxide (GO) and Selcted Area Electron Diffraction pattern.

The prepared GO was observed (Figure 2) to have a wrinkled sheet-like structure and the size of the sheet is in the nano range. TEM image also describes that the synthesized graphene oxide is amorphous in nature. The morphology of GO, comprising of thin stacked flakes of shapes and having well distinct multilayered structure at the edge, can be undoubtedly seen in Figure 2. It was anticipated that the electronegativity of oxygen atom of –OH and –COOH groups on GO layer surface eased the further oriented accumulation of cation radical of carbon. The diffraction pattern from the TEM analysis shows amorphous nature of the synthesized GO. The wrinkled structure and amorphous nature of the synthesized sample confirm the successful oxidation of graphite flakes into graphene oxide nanosheets. For the preparation of nanocomposites, it is advisable to have rough surface of GO as it will decrease the restacking tendency [32].

#### 3.1.2. Fourier Transform Infrared Spectra of Synthesized Graphene Oxide

**Figure 3:**
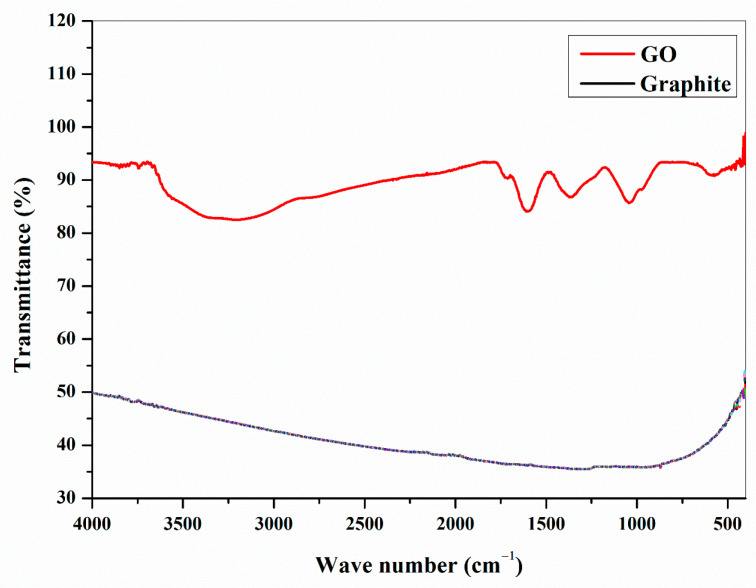
FTIR Fourier Transform Infra-Red spectra of prepared GO and Graphite.

The FTIR spectra of graphite and GO is specified in Figure 3. Peaks for a number of well-designed groups can be understood in the FTIR spectrum of GO. The various sorts of oxygen functionalities existing in GO might be seen from the spectra. Contrasted to graphite, GO illustrates new peaks at 3400 cm^−1^ which is owing to the O–H stretching vibrations, whereas at 1720 cm^−1^ it is attributed to the stretching vibrations from C=O, peak at 1600 cm^−1^ is from the skeletal vibrations of un-oxidized graphitic state. The C–OH stretching vibrations at 1220 cm^−1^ and C–O stretching vibrations at 1060 cm^−1^ also can be perceived from the FTIR spectra. By analyzing the FTIR spectra it is evident that the GO is successfully synthesized and shows all characteristic peaks especially C=O and O–H peaks.

### 3.2. Characterizations of Polymer Nanocomposites

#### 3.2.1. FTIR Spectra of Polymer Nanocomposites

**Figure 4:**
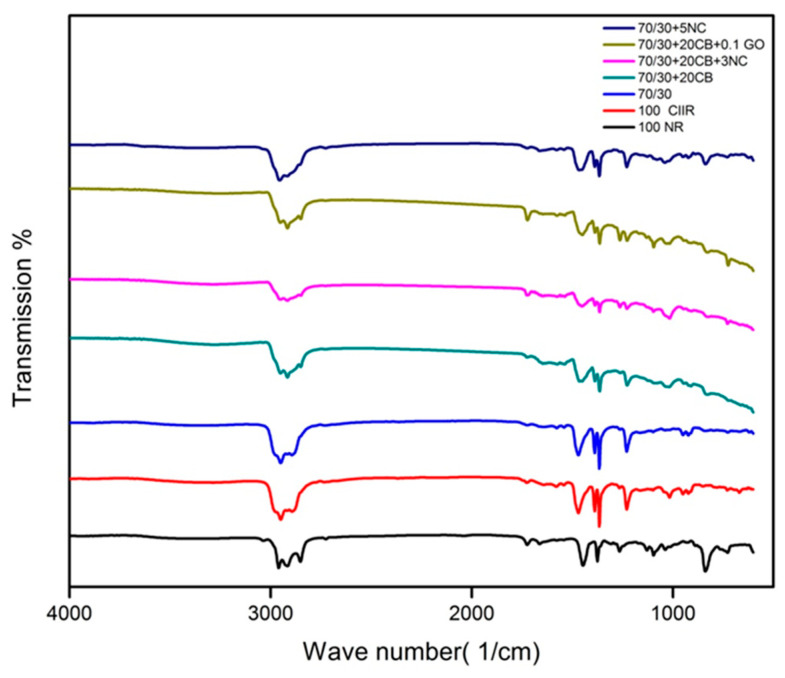
FTIR spectra of polymer nanocomposites.

The FTIR analyses give information about the role of filler in the polymer and the type of filler interactions in the blend nanocomposites. Figure 4 shows the FTIR spectra of polymer nanocomposites before vulcanization. The distinctive peaks of cis-1,4 polyisoprene, at 2960, 2927 and 2852 cm^−1^ is assigned to C–H stretching, 1661 cm^−1^ credits the presence of C=C stretching, 1448 cm^−1^ due to C–H deformation of –CH_2_–moiety, the peak at 1376 cm^−1^. On account of C–H deformation of –CH_3_ and 835 cm^−1^ owing to C–H deformation of C=C–H, could be recognized to NR molecules. The same was observed in the case of CIIR where characteristic symmetric and asymmetric stretching vibrations were observed. The CH_2_ (2875 cm^−1^) and CH_3_ (2951 cm^−1^), 2894 cm^−1^, (C–H stretching of CH_3_) Also the bending vibrations of C–H were witnessed at CH_3_: 1472 cm^−1^, CH_3_: 1388 cm^−1^, CH_3_: 1366 cm^−1^ (C–H deformation of –CH_3_). The peak differences of 100 NR and 100 CIIR is due to the chemical composure of each material. It is clear from the FTIR analysis that the hybrid filler has no chemical bonding with the rubber, it only has physical interactions. If there are no chemical interactions between filler and rubber the basic structure of the matrix is not altered it will be beneficial to end product application.

#### 3.2.2. Morphological Analysis

**Figure 5:**
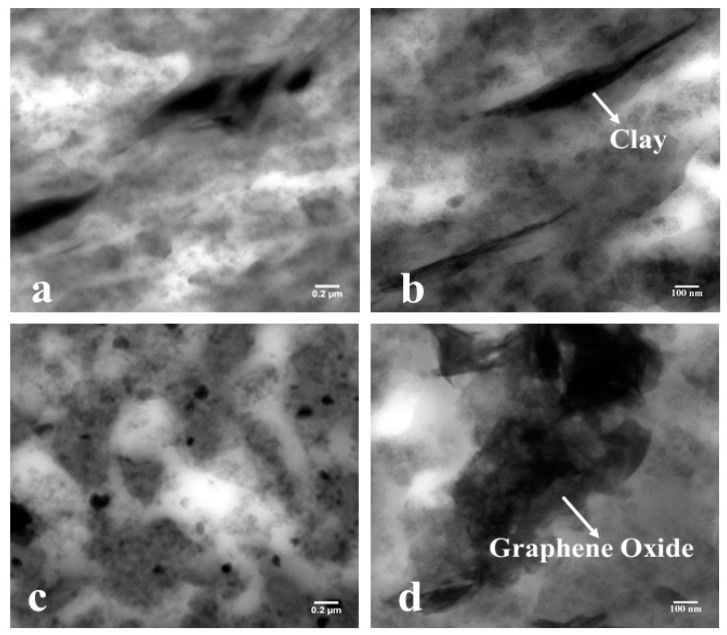
TEM images of (**a**,**b**) nanoclay in chlorobutyl rubber (CIIR)/natural rubber (NR) and (**c**,**d**) graphene oxide in CIIR/NR.

Figure 5a shows 3 phr nanoclay reinforced in CIIR/NR blend composite and the Figure 5b shows 0.1 phr graphene oxide reinforced in CIIR/NR blend composite. The TEM pictures of nanocomposites appear the nature of the scattering of nano-clays and GO within the mixes. Figure 5a appears a total scattering of nano-clay over the inspected range. The nano-clays are dispersed within the nanocomposites in the form of tiny nano-units. The researchers from different parts of the world as of now detailed these micro-units, named as nano-units, and the collected works are saying that a vigorous interaction between the elastic chains, carbon black, and the nanofillers are required to make such units. The researchers claim that the arrangement of such units can slow down the diffusion of gas atoms through the mixes. The foundation of such nano-units within the rubber nanocomposites will further uphold the interactions between the rubber mixes and nano-clays/GO. The dark portions of the TEM images imply NR phase and the bright portions entail CIIR phase of blend nanocomposites (Figure 5a,b). From Figure 5a, it is evident that nanoclay has significant interaction with both CIIR and NR matrix. But in the case of graphene oxide, the interaction is less compared to that of nanoclay due to the polar nature of graphene oxide. Even though the nanoclay platelets have more affinity towards the NR matrix, they show good dispersion in both phases leading to strong interface. The TEM images of composites further affirms the layered structure of nano-clay and graphene oxide.

#### 3.2.3. Rheological Studies

The rheological study was done at 160 °C and the data obtained are given in Table 3. Cure time is the time taken for 90% curing to occur [30]. From the cure time characteristics, it is very clear that the time taken to cure pure CIIR is very high when compared to NR. 

In the case of CIIR/NR blend it can be observed that the cure time is increased as compared to neat NR. This can be due to the incompatibility of the system as a result of the migration of curatives towards to more unsaturated NR phase. We also have to consider another important point, i.e., upon the addition CIIR to NR the overall unsaturation of the resulting component is lower than the neat NR. The factors affecting the curative migration are the degree of unsaturation, viscosity and polarity of the polymers. Curative migration occurs from highly unsaturated rubber to lower unsaturation. Additionally, the faster cure rate in highly unsaturated rubber makes the imbalance more prominent [31,32]. Here in NR, the number of unsaturation is very high compared to CIIR and curing is very fast but in CIIR the unsaturation is less as a result, CIIR takes more time to get cured. In the case of 70/30 CIIR/NR blend system; the possibility for preferential migration can occur due to the variation in polarity, viscosity and unsaturation. Additionally, the high incompatibility between CIIR/NR can reduce the interphase crosslinking thereby increasing the cure time. It is very evident that after adding the fillers the cure time decreased compared to the neat blend up to a certain limit this could be due to the cure activating result as result of a complex reaction which is already reported. However, further loading of filler marginally increases the cure time on account of the accelerator absorption by the graphene oxide surfaces. In the case of both the hybrid nanocomposites a hybrid effect can be observed. The presence of two fillers such as carbon black and nanoclay (70/30/20CB/2NC) and carbon black and graphene oxide (70/30/20CB/0.2GO) composition reduces the agglomeration and facilitate the crosslinking thereby reducing the cure time.

### 3.3. Mechanical Properties

**Figure 6:**
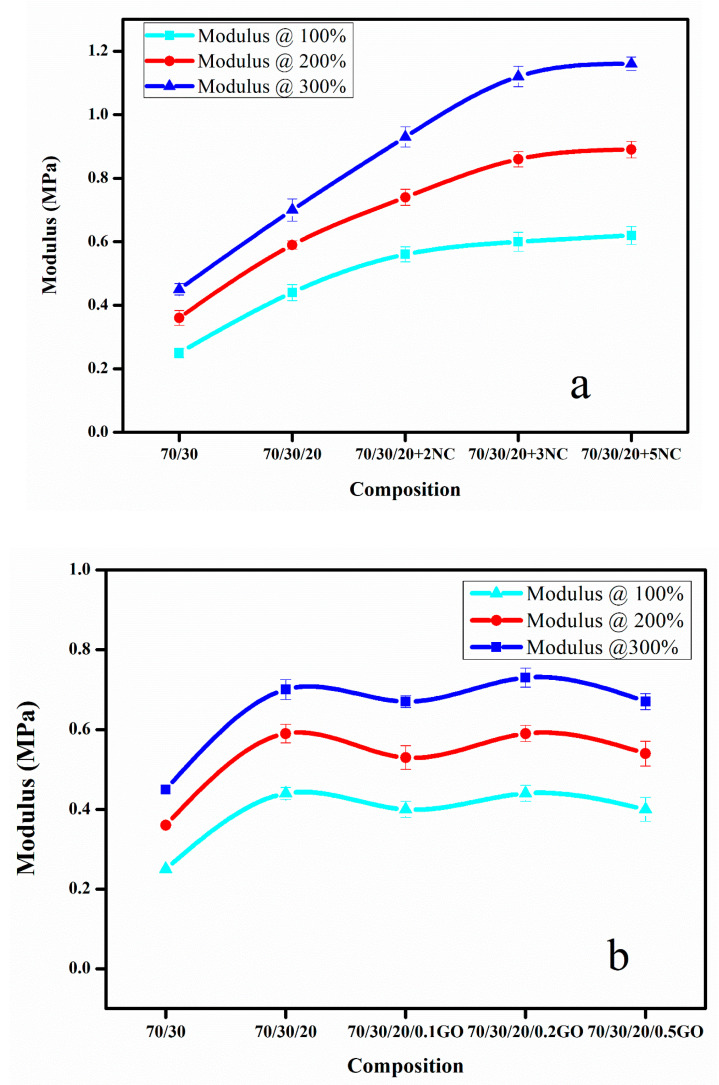
(**a**,**b**) Effect of nanoclay and graphene oxide on modulus of prepared nanocomposites at 100%, 200% and 300% elongation.

**Figure 7:**
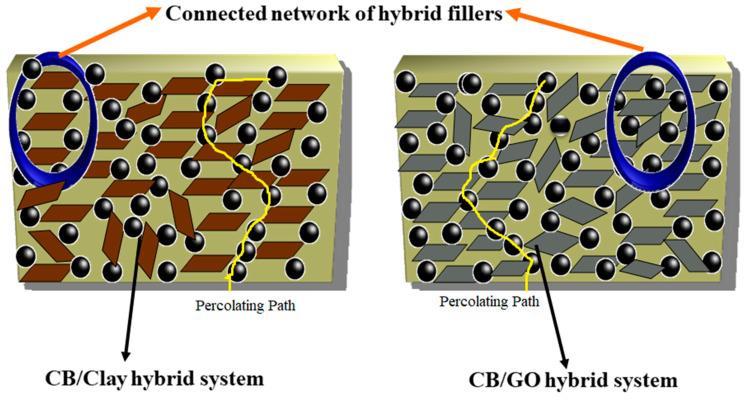
Schematic representation showing the interaction of GO and nanoclay with carbon black (CB) and the difference in dispersion of both the fillers in presence of CB.

The Figure 6a,b shows modulus of blend nanocomposites with different loading of nanoclay and GO at 100, 200 and 300% elongation. The moduli of the nanocomposites were found to increase on adding the nanofiller (Figure 6a,b). The effect was more prominent in the case of nanoclay. This may be due to the greater number of polymer–filler interactions than filler–filler interactions. The slightly polar nature of nanoclay and the presence of rich alkyl groups in nanoclay help to create an interaction with both CIIR and NR matrix when compared to GO. Graphene oxide is highly polar in nature due to the presence of oxygen functionalities, so it has better interaction with polar CIIR [33]. The presence of CB should have a profound effect on improving the dispersion of nanofillers in the rubber matrix. It reduces the possibilities of filler–filler interaction. From Figure 7, it is clear that the effect of CB in dispersing the nanofiller is more efficient in the case of nanoclay. This can be due to the relatively less polar–polar interaction between the nanoclay compared to GO–GO interaction which is having strong polar nature. The elongation at break for composites with both nanofiller shows a decrease with increase in filler loading. This is due to the restriction imparted by filler to the flexibility. The CB plays a major role to disperse the GO and nanoclay in the CIIR/NR matrix (Figure 7), it is also very effective in preventing the restacking or agglomeration tendency of both the nanofillers thereby increases the possibility of better polymer–filler interactions.

### 3.4. Gas Permeability Study

**Figure 8:**
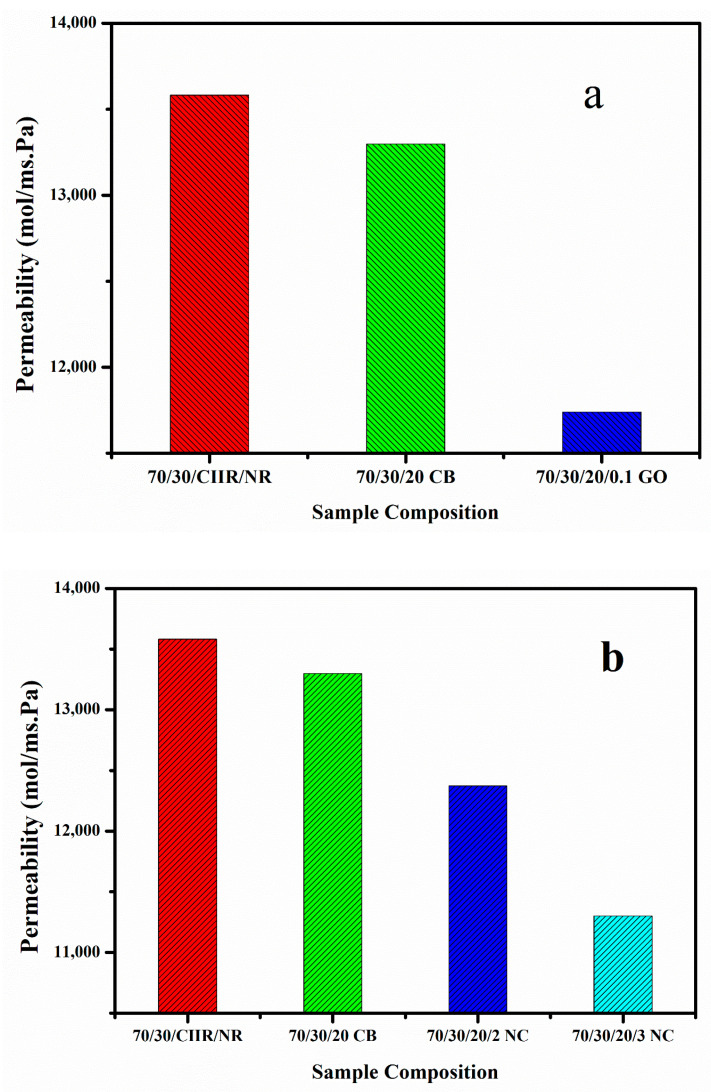
(**a**,**b**) Gas permeability values of CB/GO and CB/nanoclay hybrid nanocomposite.

The permeability of the prepared composites was studied using oxygen as per the ASTM D1434-82 standard. It was observed that the permeability values of the CIIR/NR blend nanocomposites with CB is much lower than that of blend with CIIR/NR (Figure 8a). When we add 0.1 phr of GO to CIIR/NR/CB blend the permeability of air again decreases drastically, this is because of the hybrid filler effect of CB/GO [34,35]. GO creates an effective tortuous path for oxygen to permeate through the rubber matrix. CB also plays an important role in preventing the restacking or agglomeration nature of GO. In fact, CB effectively interacts with GO and reduces the filler–filler interaction as well as increases the filler–matrix interaction. 

At the same time by the addition of nanoclay to the CIIR/NR/CB blend, we can observe the same decreasing trend in air permeability up to 3 phr loading, at higher loading the effect of hybrid nanofillers in lowering the permeability of the air is poor due to the agglomeration of nanofillers, (Figure 8b). The reduction in permeability of air at lower loading can be attributed to the better dispersion of nanofillers and the tortuous path created by them and also the added CB is enough to reduce the filler–filler interaction. The effect of hybrid filler in reducing the oxygen permeability values could be clearly established through this. The gas permeability value of the blend decreased after the addition of layered fillers such as graphene oxide and nanoclay. A 14% and 9% decrease in permeability values are observed for 70/30/20/0.1 GO and 70/30/20CB/2NC respectively.

**Figure 9:**
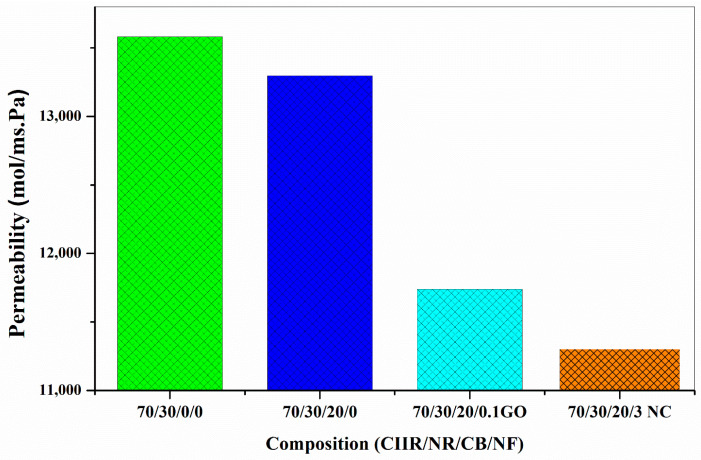
Comparison of permeability values of CB/GO and CB/nanoclay nanocomposite.

On comparing the results obtained for GO and nanoclay, It is well established that the interaction of CB and nanoclay is more effective when related to the interaction of CB and GO (Figure 9). The permeability values also show that CB/nanoclay hybrid filler is very effective due to the better interaction between them.

## 4. Conclusions

CIIR/NR blend was prepared at 70/30 composition and the effect of hybrid filler was studied using CB/NC, as well as CB/GO. The GO used was synthesized via modified hummer’s method, characterized using FTIR and TEM analysis and confirmed the formation of pristine graphene oxide. Rheological study of CIIR/NR polymer blend nanocomposites indicates that cure time decreases at filler loading. The prepared nanocomposites show an increase in modulus when GO and nanoclay are added to the CIIR/NR matrix along with CB. The composites with CB and nanoclay show a gradual increase in modulus with filler loading while in the case of CB/GO there is no regular trend in the modulus of the composite. Addition of optimum amount of nanoclay or graphene oxide in CIIR/NR blend can improve the gas impermeability of the system due to the tortuous path created by the layered structure of nanofillers with the help of CB. The interaction of CB and dispersion of nanofillers into the matrix is very effective in the case of nanoclay compared to GO. Such materials find potential applications in tubeless tyres and other gas-impermeable appliances like masks and sports utilities. However, it is also observed that the higher loading of fillers reduces its overall gas impermeability property due to the agglomeration of nanofillers. Thus, adding nanofillers to the CIIR/NR blend can help to improve the properties and can obtain the polymer nanocomposites with the desired combination of properties.

## Figures and Tables

**Table 1:** Compounding materials used for the preparation of CIIR/NR blend nanocomposites. * Phr—parts per hundred rubber by weight.

Materials	Amount (Phr) *
Curing Agent (Phenol Formaldehyde resin)	6
Homogenizing Agent (Struktol 40 MS)	6
Zinc oxide	3
Stearic Acid	1
MBTS (Accelerator)	1.25
Sulphur	0.5

**Table 2:** The formulations used for the preparation of polymer nanocomposites. * Phr—parts per hundred rubbers by weight.

Sl. No.	CIIR *	NR *	CB *	NC *	GO *
1	100	-	-	-	-
2	-	100	-	-	-
3	70	30	-	-	-
4	70	30	20	-	-
5	70	30	20	2	-
6	70	30	20	3	-
7	70	30	20	5	-
8	70	30	20	-	0.1
9	70	30	20	-	0.2
10	70	30	20	-	0.5

**Table 3:** Cure characteristics of polymer nanocomposites.

Sl No.	Samples	Cure Time (min:s)
1	100 CIIR	11:37
2	100 NR	5:47
3	70/30 CIIR/NR	17:26
4	70/30/20CB	16:33
5	70/30/20CB/2NC	14:33
6	70/30/20CB/3NC	16:20
7	70/30/20CB/5NC	16:57
8	70/30/20CB/0.1GO	15:16
9	70/30/20CB/0.2GO	14:40
10	70/30/20CB/0.5GO	15:16

## Data Availability

Data can be available upon request from the authors.
